# Case report: novel *PCDH15* variant causes usher syndrome type 1F with congenital hearing loss and syndromic retinitis pigmentosa

**DOI:** 10.1186/s12886-022-02659-6

**Published:** 2022-11-16

**Authors:** Nelson Chen, Hane Lee, Angela H. Kim, Pei-Kang Liu, Eugene Yu-Chuan Kang, Yun-Ju Tseng, Go Hun Seo, Rin Khang, Laura Liu, Kuan-Jen Chen, We-Chi Wu, Meng-Chang Hsiao, Nan-Kai Wang

**Affiliations:** 1 Department of Ophthalmology, Edward S. Harkness Eye Institute, Columbia University Medical Center, 635 West 165th Street, New York, NY 10032 USA; 2 Faculty of Health Sciences, Queen’s University, Kingston, Ontario Canada; 3 Division of Medical Genetics, 3billion, Inc., Seoul, South Korea; 4 College of Medicine at the State University of New York at Downstate Medical Center, Brooklyn, NY USA; 5 Department of Ophthalmology, Kaohsiung Medical University Hospital, Kaohsiung Medical University, Kaohsiung, Taiwan; 6 School of Medicine, College of Medicine, Kaohsiung Medical University, Kaohsiung, Taiwan; 7 Institute of Biomedical Sciences, National Sun Yat-sen University, Kaohsiung, Taiwan; 8 Department of Ophthalmology, Chang Gung Memorial Hospital, Linkou Medical Center, Taoyuan, Taiwan; 9 College of Medicine, Chang Gung University, Taoyuan, Taiwan; 10 Graduate Institute of Clinical Medical Sciences, College of Medicine, Chang Gung University, Taoyuan, Taiwan; 11 School of Traditional Chinese Medicine, Chang Gung University, Taoyuan, Taiwan; 12 Department of Pathology and Cell Biology, Columbia University Medical Center, New York, NY USA; 13 Vagelos College of Physicians and Surgeons, Columbia University, New York, USA

**Keywords:** Usher syndrome type 1F (USH1F), *PCDH15*, Protocadherin-15, Loss of function, Nonsense-mediated decay, Syndromic retinitis pigmentosa, Congenital hearing loss, Case report

## Abstract

**Background:**

Usher syndrome (USH) is an autosomal recessive disorder primarily responsible for deaf-blindness. Patients with subtype Usher syndrome type 1 (USH1) typically experience congenital sensorineural hearing loss, abnormal vestibular function, and retinitis pigmentosa (RP). Here we present a case of Usher syndrome type 1F (USH1F) with a novel homozygous variant in the calcium-dependent cell-cell adhesion protocadherin-15 (*PCDH15*) gene.

**Case presentation:**

Ophthalmic examinations were evaluated over a course of 10 years and the disease-causing variant was identified by whole exome sequencing (WES). Initial and follow-up examination of color fundus photos after 10 years revealed an increase in bone spicule pigment deposits in both eyes. A parafoveal hyper-AF ring in both eyes was shown in fundus autofluorescence (FAF) with a progressive diameter-wise constriction observed over 8 years. Outer nuclear layer (ONL) loss was observed in parafoveal and perifoveal regions of both eyes on spectral domain–optical coherence tomography (SD-OCT). Full-field electroretinography (ffERG) showed extinguished global retinal function. WES identified a novel two-base-pair deletion, c.60_61del (p.Phe21Ter), in the *PCDH15* gene, confirming the diagnosis of USH1F.

**Conclusions:**

We report a novel homozygous *PCDH15* pathogenic variant expected to lead to nonsense-mediated decay (NMD) of *PCDH15* mRNA. The patient exhibits a loss of function with USH1F, experiencing congenital hearing loss and syndromic RP.

**Supplementary Information:**

The online version contains supplementary material available at 10.1186/s12886-022-02659-6.

## Background

Usher syndrome (USH) is an autosomal recessive disorder that is widely responsible for deaf-blindness. This genetic disorder was first reported in 1914 by Charles Usher, who described 69 patients from 40 separate families presenting with retinitis pigmentosa and hearing loss (Usher, 1914). Usher syndrome is a ciliopathy that disrupts photoreceptor ciliogenesis in the retina and kinocilia in the inner ear [1]. Patients with USH are characterized by rod-cone dystrophy, partial-complete sensorineural hearing loss, and a possibility of vestibular dysfunction [2]. The severity and onset of symptoms depend on the clinical type — Usher type 1 (USH1), attributed to variants in protein coding genes *USH1C, CDH23, PCDH15,* and *USH1G*; Usher type 2 (USH2) from variants in *USH2A, ADGRV1,* and *WHRN*; Usher type 3 (USH3) from *CLRN1* [3]; and atypical Usher syndrome type 4 (USH4) from *ARSG* [4].

Variants in *PCDH15* are responsible for 11–19% of USH1 cases and is categorized specifically as Usher type 1F (USH1F, OMIM: 602083). USH1F is typically described by congenital sensorineural hearing loss, abnormal vestibular function, and the prepubertal onset of progressive retinitis pigmentosa (RP) [2]. The *PCDH15* gene spans a genomic region of 980 kb composed of 33 exons. It encodes for the protocadherin-15 protein, belonging to integral membrane proteins that mediate calcium-dependent cell-adhesion, which play a crucial role in retinal and cochlear function [3].

In this report, we present a case of USH1F with a novel homozygous variant c.60_61del in the *PCDH15* gene (NM_001384140.1), creating a stop codon (p.Phe21Ter) after the 20th amino acid.

## Case presentation

A 21-year-old East-Asian female presented to our clinic with peripheral vision loss and impaired night vision. Past medical history included congenital hearing loss, for which cochlear implant surgery was done in her early childhood. The patient is the product of a reportedly nonconsanguineous union between healthy parents and had no pertinent family history. The patient reported an unaffected brother and no history of smoking and drinking.

**Fig. 1:**
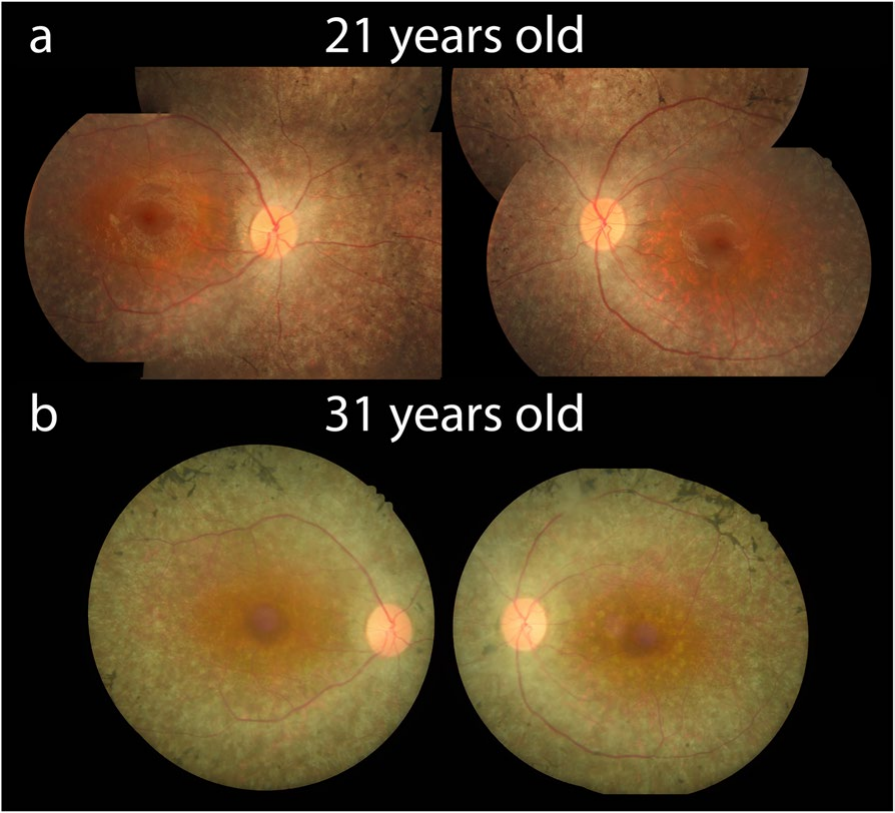
Color fundus of both eyes. **a** At presentation, fundus shows mild bilateral retinal vessel attenuation and bone spicule deposits in both eyes. **b** Follow up fundus reveals a pale retina with a progression in bilateral retinal vessel attenuation and amount of bone spicule pigment deposits

At the time of presentation, the patient displayed a best-corrected visual acuity (BCVA) of 20/50 and 20/40 in the right eye and left eye, respectively. Initial color fundus images revealed mild, bilateral retinal vessel attenuation and bone spicule pigment deposits in the mid-periphery of both eyes (Fig. 1a).

**Fig. 2:**
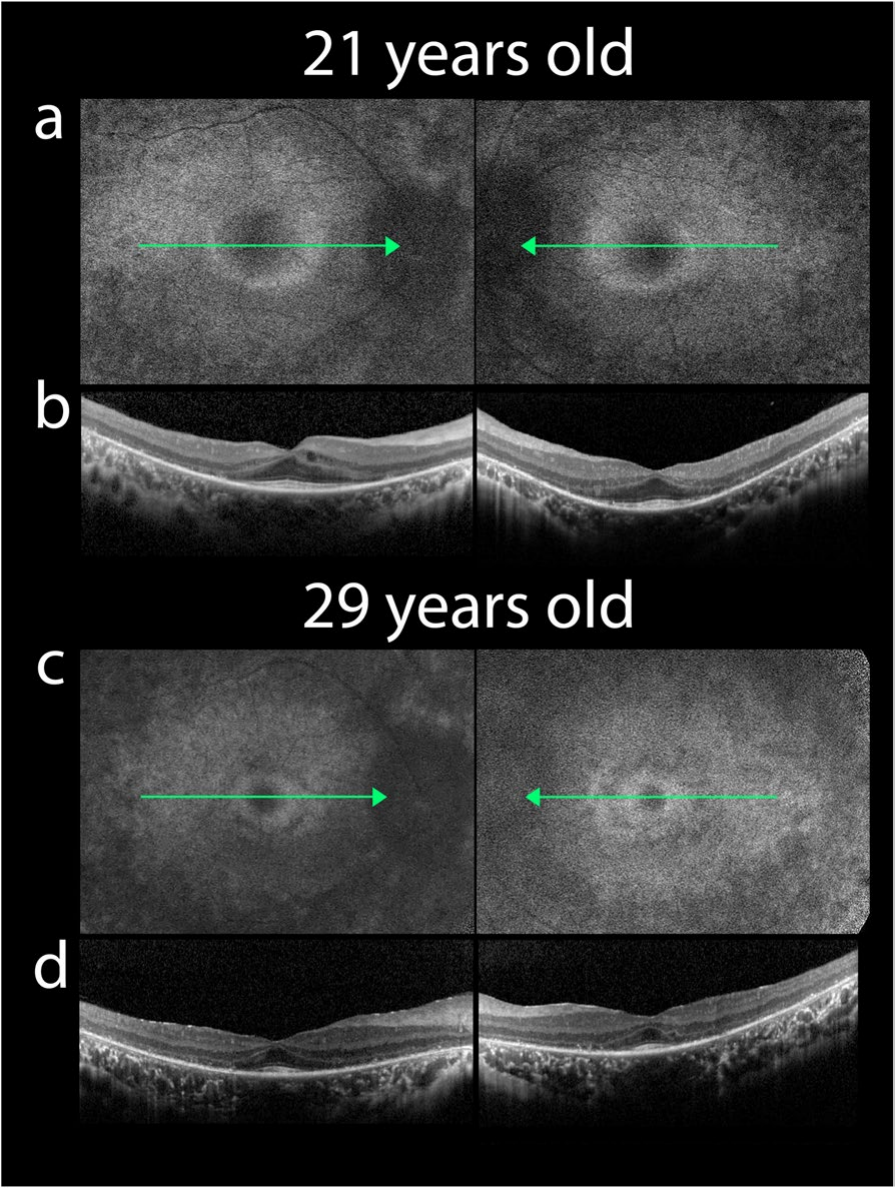
Initial fundus autofluorescence (FAF) and spectral domain optical coherence tomography (SD-OCT) of both eyes. **a** FAF at presentation reveals a parafoveal hyper-AF ring and in both eyes. **b** SD-OCT at presentation shows outer nuclear layer (ONL), external limiting membrane (ELM), and inner segment ellipsoid (ISe) loss in the parafoveal and perifoveal regions in both eyes. **b** Cystoid macular edema in right eye can be observed in parafoveal region. Follow-up fundus autofluorescence (FAF) and spectral domain optical coherence tomography (SD-OCT) images of both eyes. **c** Follow up FAF shows a constriction in the parafoveal hyper-AF ring diameter. **d** Follow up SD-OCT shows an equivalent shortening in ELM and ISe bands in both eyes with additional reduction in ONL thickness in both eyes

Fundus autofluorescence (FAF) images revealed an abnormal parafoveal hyperautofluorescence (hyper-AF) ring, surrounded by hypo-AF spots in both eyes (see Table 1 and Fig. 2a). Initial spectral domain-optical coherence tomography (SD-OCT) images revealed the preservation of the external limiting membrane (ELM) and inner segment ellipsoid (ISe) band in only the foveal region. Significantly reduced outer nuclear layer (ONL) thickness in the parafoveal and perifoveal area suggested heavy photoreceptor degeneration (Fig. 2b). Cystoid macular edema was observed in the inner nuclear layer (Fig. 2b). The aligned FAF and SD-OCT images revealed that the outer border of the hyper-AF ring aligns with the point of ELM disruption. These observations correspond with previous assessments of hyper-AF ring structure in patients with RP [5]. Full-field electroretinogram (ffERG) tests displayed an extinguished rod response, combined rod-cone response, cone response, and 30 Hz flicker response in both eyes since the time of presentation (Supplementary Fig. 1).

**Table 1:** Progression of quantitative clinical observations over 10 years

	Baseline		8-year follow-up		10-year follow-up	
	OD	OS	OD	OS	OD	OS
**BCVA**	20/50	20/40			20/40	20/40
**Hyper-AF Ring Diameter (μm)**^**a**^	3048	2946	1727	1524		

*BCVA* best-corrected visual acuity, *Hyper-AF* hyperautofluorescence ring, *OD* right eye, *OS* left eye.

^a^Horizontal diameter

Follow-up examinations were conducted over a course of 10 years. At 31-years of age, the patient’s BCVA remained consistent to initial measurements at presentation (see Table 1 for details). However, the patient’s fundus image shows a progression in bilateral retinal vessel attenuation and retinal depigmentation, along with an increased amount of bone spicule pigment deposits in the mid-periphery region, extending towards the near-peripheral retina (Fig. 1b).

Follow-up FAF and SD-OCT images show a progressive diameter-wise constriction in the perifoveal hyper-AF ring (see Table 1 and Fig. 2c), with an equivalent shortening in ELM and ISe bands in both eyes (Fig. 2d). An additional reduction in ONL thickness was also observed in the follow-up SD-OCT images in both eyes (Fig. 2d).

The patient was diagnosed with Usher syndrome type 1 based on the presented characteristics of congenital hearing loss and syndromic RP. Subtype USH1F was confirmed by whole exome sequencing (WES) that revealed a previously unseen homozygous variant, c.60_61del in the *PCDH15* gene (NM_001384140.1), which is predicted to create a stop codon (p.Phe21Ter) after the 20th amino acid. The variant was further confirmed by Sanger sequencing (Supplementary Fig. 2).

## Discussion and conclusions

The patient presenting with RP and congenital hearing loss was monitored over a course of 10 years. Progression in RP was observed with an increase in bone spicule deposits, a constriction of the perifoveal hyper-AF ring, and a reduction in ONL thickness. Since *PCDH15* is abundantly expressed in both rod and cone photoceptors [6], the patient’s clinical progression indicates significant photoreceptor degeneration associated with the novel two-base-pair deletion (c.60_61del) in *PCDH15* (NM_001384140.1). This variant is predicted to produce a premature stop codon in exon 2/33, which is expected to result in nonsense-mediated decay (NMD) of *PCDH15* mRNA. The impaired expression of *PCDH15* in rod and cone photoreceptors is likely associated with the patient’s progression in RP with a loss in rod photoreceptors and secondary cone degeneration. Although the exact molecular function of retinal protocadherin-15 is ambiguous [7], using existing animal models with *PCDH15* variants may help further understand the patient’s clinical observations. A similar founder variant in *PCDH15* that is unique to Ashkenazi Jews, p.Arg245Ter, was simulated using a *Pcdh15*^*R250X*^ knockin mutant mouse model that phenocopied human p.Arg245Ter congenital hearing loss and abnormal vestibular function [7]. While present in wild-type mice, immunostaining revealed the absence of protocadherin-15 expression in the inner segments of photoreceptors, outer plexiform layer, the ganglion cell layer, and retinal pigment epithelium (RPE) in *Pcdh15*^*R250X*^ mice. Under photopic conditions, the loss of protocadherin-15 hinders the transportation of arrestin and transducin between the photoreceptor outer segment (OS) and inner segment (IS) to desensitize or bind to opsin, respectively. This results in abnormal protein localization in the phototransduction cascade and retinoid cycle [7]. Additionally, a reduction in enzymes CRALBP and RPE65 consequently reduced 11-cis-retinal functions [7]. Combined with the gross retinal degeneration observed in the patient, both observations may help explain extinct ffERG amplitudes in Supplementary Fig. 1. However, acute retinal degeneration was less severe in mice when compared to human pathophysiology [7]. This may be attributed to the absence of protocadherin-15 associated calyceal processes in rodent photoreceptor cells that are present in humans, frogs, and monkeys [8]. Knockdown *PCDH15* frog models show the degeneration and loss of photoreceptor function due to the proposed role of the calyceal process in rod and cone maintenance and development [8].

The c.60_61del variant was identified as homozygous. Although the parents were not known to be related to each other, the patient had regions of homozygosity (ROH) across ~ 0.8% of the genome suggesting that the parents are distantly related. The *PCDH15* homozygous variant was found within one of the larger ROH (~ 10 Mb). Therefore, even though the parents were not available for Sanger sequencing, it is likely that each parent is heterozygous for the variant. There was no evidence of a large copy-number-loss variant spanning the *PCDH15* gene from the WES data.

Loss of function variants are known to be the disease-causing mechanism with many pathogenic variants reported in the literature and databases being null variants. Clinical observations reported a loss of function in the patient’s phenotype, which may be attributed to NMD mechanisms eliminating mRNA containing a premature termination codon [9]. Since the variant is located at the beginning of the *PCDH15* gene on exon 2/33, nonsense-mediated decay of *PCDH15* mRNA is expected to occur. However, if a small amount of mRNA does escape the NMD pathway, then the shortened peptide (around 21 amino acids), will most likely be degraded. Hence, it is likely that no protein products are produced. To further examine the mechanisms that relate to a loss of function, future animal models may use qPCR techniques to determine the expression of *PCDH15* mRNA to examine the role of NMD in this novel variant.

In this longitudinal report, we followed a patient with a novel variant in the *PCDH15* gene over a course of 10 years. A novel nonsense variant c.60_61del results in typical USH1F clinical symptoms, such as congenital hearing loss and progressive RP [2]. Although the patient was first examined in her twenties, with clinical features resembling clinical subtypes of USH 2 and USH 3 [2], her ERG responses (Supplementary Fig. 1) resemble RP in the advanced stage [10] suggesting a prepubertal onset of photoreceptor degeneration, which correspond with bilateral ONL thinning in SD-OCT (Fig. 2d). Thus, ffERG is essential for an early and correct diagnosis of USH1 when combined with the presence of congenital hearing loss, allowing clinicians to test genes related to USH1.

## Supplementary Information

**Additional file 1: Supplementary Fig. 1.** Full-field electroretinography (ffERG) at presentation shows extinguished rod response, combined rod-cone response, cone response and 30 Hz flicker response in both eyes.

**Additional file 2: Supplementary Fig. 2.** Sanger sequencing of the *PCDH15* gene*.* The sequence trace shows the *PCDH15* variant, which is consistent with whole exome sequencing (WES) test results.

## Data Availability

The dataset that used and analyzed during the current study are available from the corresponding author on reasonable request.

## Abbreviations

USH: Usher syndrome
USH1: Usher syndrome type 1
USH2: Usher syndrome type 2
USH3: Usher syndrome type 3
USH4: Usher syndrome type 4
USH1F: Usher type 1F
BCVA: Best-corrected visual acuity
FAF: Fundus autofluorescence
SD-OCT: Spectral domain-optical coherence tomography
ELM: External limiting membrane
ISe: Inner segment ellipsoid
ONL: Outer nuclear layer
ffERG: Full-field electroretinogram
WES: Whole exome sequencing
NMD: Nonsense-mediated decay
RPE: Retinal pigment epithelium
OS: Outer segment
IS: Inner segment
ROH: Regions of homozygosity
