# Supplementary figures and images for: Case report: novel PCDH15 variant causes usher syndrome type 1F with congenital hearing loss and syndromic retinitis pigmentosa
Source: BMC Ophthalmol. 2022 Nov 16;22:441. doi: 10.1186/s12886-022-02659-6 (PMC9670441; doi:10.1186/s12886-022-02659-6)

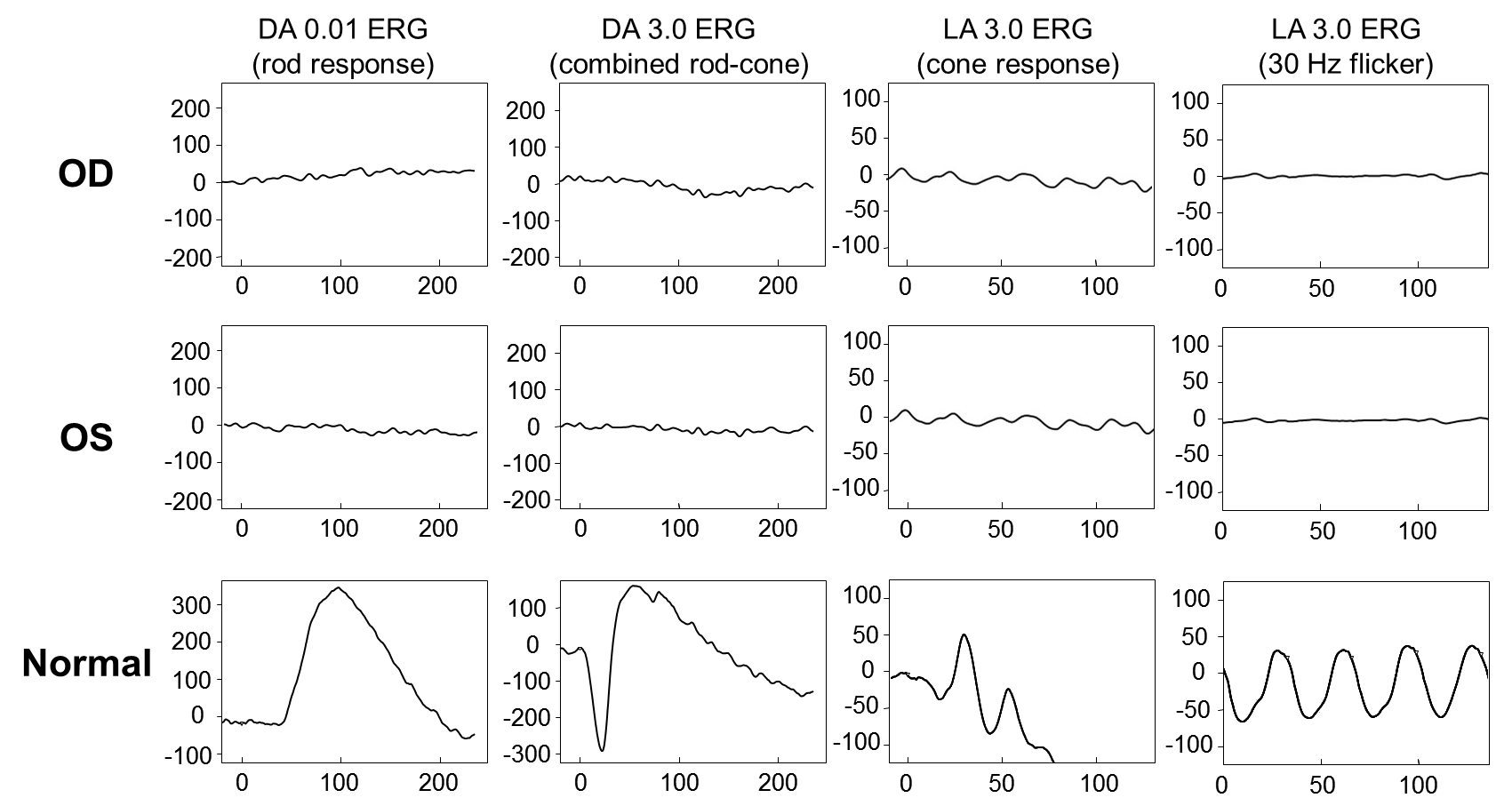

Supplement: Supplementary file 1 — Additional file 1: Supplementary Fig. 1. Full-field electroretinography (ffERG) at presentation shows extinguished rod response, combined rod-cone response, cone response and 30 Hz flicker response in both eyes. [file 12886_2022_2659_MOESM1_ESM.tif]

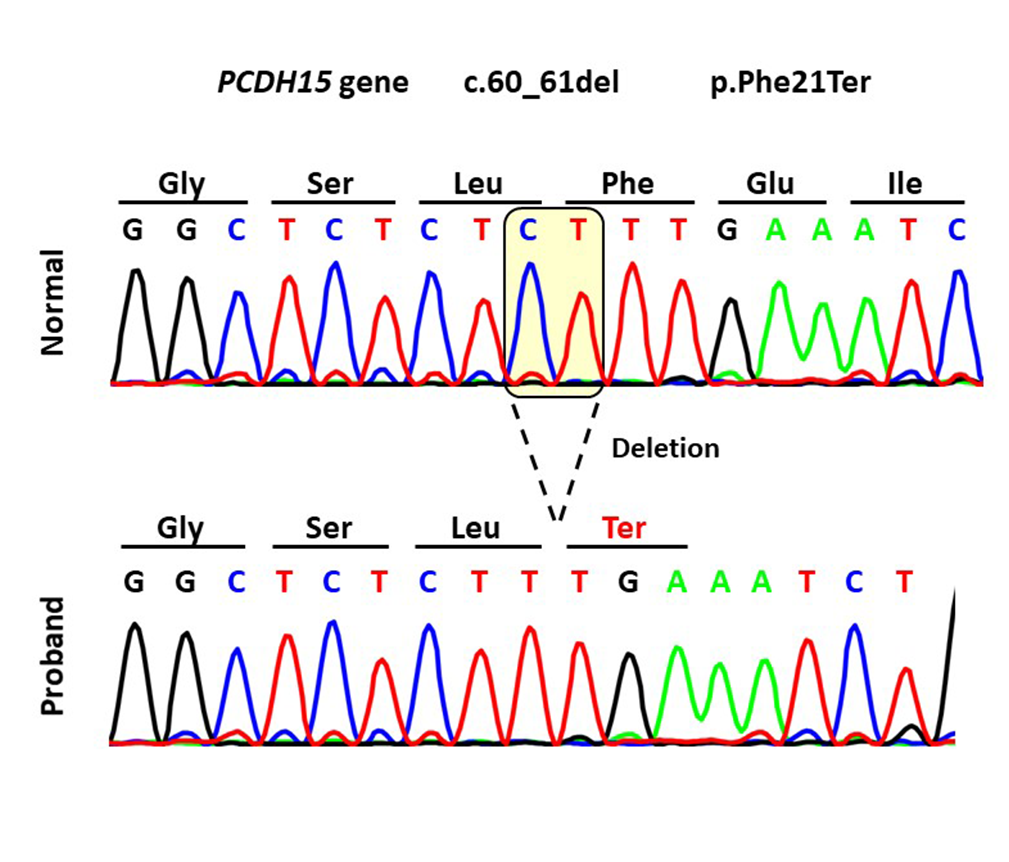

Supplement: Supplementary file 2 — Additional file 2: Supplementary Fig. 2. Sanger sequencing of the PCDH15 gene. The sequence trace shows the PCDH15 variant, which is consistent with whole exome sequencing (WES) test results. [file 12886_2022_2659_MOESM2_ESM.tif]
